# Difference of brain-derived neurotrophic factor expression and pyramid cell count during mastication of food with varying hardness

**DOI:** 10.1590/1678-7757-2018-0182

**Published:** 2019-04-01

**Authors:** Jenny Sunariani, Christian Khoswanto, Wahyuning Ratih Irmalia

**Affiliations:** 1 Universitas Airlangga, Faculty of Dental Medicine, Department of Oral Biology, Surabaya, Indonesia.; 2 Universitas Airlangga, Faculty of Dental Medicine, Surabaya, Indonesia.

**Keywords:** Hippocampus, Mastication, Pyramidal cell, BDNF

## Abstract

Previous studies suggested that mastication activity can affect learning and memory function. However, most were focused on mastication impaired models by providing long-term soft diet. The effects of chewing food with various hardness, especially during the growth period, remain unknown.

**Objective:**

To analyze the difference of hippocampus function and morphology, as characterized by pyramidal cell count and BDNF expression in different mastication activities.

**Materials and Methods:**

28-day old, post-weaned, male-Wistar rats were randomly divided into three groups (n=7); the first (K0) was fed a standard diet using pellets as the control, the second (K1) was fed soft food and the third (K2) was fed hard food. After eight weeks, the rats were decapitated, their brains were removed and placed on histological plates made to count the pyramid cells and quantify BDNF expression in the hippocampus. Data collected were compared using one-way ANOVA.

**Results:**

Results confirmed the pyramid cell count (K0=169.14±27.25; K1=130.14±29.32; K2=128.14±39.02) and BDNF expression (K0=85.27±19.78; K1=49.57±20.90; K2=36.86±28.97) of the K0 group to be significantly higher than that of K1 and K2 groups (p<0.05); no significant difference in the pyramidal cell count and BNDF expression was found between K1 and K2 groups (p>0.05).

**Conclusion:**

A standard diet leads to the optimum effect on hippocampus morphology. Food consistency must be appropriately suited to each development stage, in this case, hippocampus development in post-weaned period.

## Introduction

Mastication is the process of crushing food and mixing it with saliva to form a bolus prior to swallowing, being a complex process involving the rhythmical opening and closing of the jaw, the secretion of saliva and tongue movement.^1^ Previous studies found correlation between mastication and the functions of other body parts aside from the digestive system. In addition to influencing nutritional intake, masticatory disorders have been proven to exert influence in increasing the risk of obesity and hyperglycemia, as well as impeding stress hormone regulation, learning processes and memory function.^2^ Mastication activity is critical as one of the sources of sensory input into the hippocampus.^3^ A decline in masticatory activity caused by a disorder can lead to hippocampal morphological changes, such as the decrease in pyramid cell count, in dendritic spines in cornu amonis-1 (CA1) and in proliferating cells in both the dentate gyrus and the astrocyte hypertrophy.^4^ Other studies also found that reduced muscle activity may decrease blood supply to the brain, thus impeding cerebral metabolism.^5^ ^,^ ^6^

The correlation between mastication activity and hippocampus morphology was identified from the neural pathway. Somatosensory input from the masticatory system may influence the hippocampus through synaptic projection from the thalamus and cerebral cortex.^3^ Brain Derived Neurotrophic Factor (BDNF) is fundamental in hippocampal learning and memory function.^7^ BDNF is a member of the neurotrophin family that promotes neuron survival, growth and plasticity, being abundantly found in the hippocampus.^8^

Previous studies observing the correlation between mastication, stress and cognitive function have proved that a long-term soft diet during the post-weaning period could lead to learning and memory impairment. On the other hand, forced mastication in mice resulting from a hard diet can increase the proliferation and survival rate of neuron cells in dentate gyrus.^7^ ^,^ ^9^ ^-^ ^12^ In the growth period, the maturation of motoric function occurs during the maturation of the entire central nervous system, as well as of the morphological and functional maturation of the craniofacial complex. Newborn mammals obtain nutritional intake by sucking, which will later develop into a masticatory motion. Regarding rats, the first mastication movements were recorded on day 12 post-natal and completely developed between days 18 and 21.^13^ Nevertheless, the effects of mastication on learning and memory functions of the hippocampus, especially during the growth period, are not fully understood.

Based on a previous study by Irmalia, et al.^14^ (2017), spatial memory tests were conducted on 28-day old, post-weaned rats after they had been fed food of varying hardness. The results confirmed that the standard diet group produced the best performance on a radial eight arm maze test, compared to the soft and hard diet groups.^14^ Thus, this study aims to analyze the differences of hippocampus function and morphology, through cellular and molecular examination of this under varying mastication activity. Based on the previous study, it is hypothesized that, providing a standard diet during the growth period will give the best response toward hippocampus morphology, compared to providing soft and hard diets.

## Material and methods

### Ethical approval

This study was approved by the Research Ethics Committee with certificate number 023/HRECC.FODM/II/2017.

### Experimental design

This was an analytic experimental study incorporating a post-test only control group design.

### Animal

Twenty-one post-weaned, male Wistar Rats (*Rattus novergicus*), aged 28 days and weighing between 80 and 100 grams were purchased from stockbreeder Bintang Jaya (Surabaya, Indonesia). Sample size was calculated using Lemeshow's formula and based on the result of the previous study.^14^ Research subjects were randomly divided into three groups (n=7): a control group (K0) fed standard rodent chow (Gemuk A, PT. Japfa Comfeed, Jakarta, DKI Jakarta, Indonesia) (Nutritional content: water 12%, protein 16%, fat 7%, fiber 8%, calcium 1%, and phosphor 0.8%), a soft diet group (K1) fed powdered seed and grain and a hard diet group (K2) fed whole seed and grain (Hamsfood, Canada) (Nutritional content: protein 12%, fat 7%, fiber 10%, calcium 0.20%, phosphor 0.30%, salt 0.08%, manganese 55 mg/kg, zinc 60 mg/kg, iron 70 mg/kg, iodine 0.6 mg/kg, selenium 0.07 mg/kg, vitamin A 7500 IU/kg, and vitamin D3 1500 IU/kg); all groups were given 20 mg food/day. Rats were acclimatized for seven days prior to treatment, subsequently being placed in a plastic cage containing wood shavings bedding covered with woven wire. They were kept at room temperature following a consistent 12-hour light-dark cycle. After eight weeks, the rats were euthanized through an anesthesia overdose and their brains were isolated.

### Histological examination

Histological examination consists of pyramidal cell count and BDNF expression, which were done using a cell counter combined with manual counting in case a system error occurred. To prevent bias, the study outcome measurement was done by an expert in histology who was blind to group allocation.

### Pyramidal cell count

Isolated brains were soaked in 10% neutral buffered formalin for two hours in individual pots. The brains were then immersed in formaldehyde, soaked for 24 hours and dehydrated using ethanol (at 70% concentration for 24 hours followed by 90% for 1 hour), rinsed using xylene and, finally, embedded in paraffin. A coronal section (5 µm) was performed following the bregma (−3.3 mm to −3.8 mm posterior to the bregma) using a microtome (Accu-Cut SRM, Sakura, AJ Alphen aan den Rijn, Netherlands).^15^ The sectioned tissues were deparaffinized, placed on object glass and stained using hematoxylin eosin (HE).^16^ Pyramidal cell count was conducted using a light microscope (Nikon H600L, Melville, New York, USA) at 400x magnification. Cell count and pictures were taken of five different fields that were randomly chosen by the system^17^ using a 300-megapixel DS Fi2 digital camera and a Nikon Image System image processor (Nikon, Melville, New York, USA).

### BDNF expression

Paraffinized brain tissue was cut into coronal sections following the bregma (−3.3 mm to −3.8 mm posterior to the bregma) using a 5 µm thick microtome (Accu-Cut SRM, Sakura, AJ Alphen aan den Rijn, Southern Netherland, Netherland). Sectioned tissues were dipped in warm water (20° to 30°C), placed on a poly-L-Lysine object glass surface and dried on a hotplate. Each plate was soaked in Xylol I, II, III for 3 minutes and then in absolute alcohol I & II for additional 3 minutes. Following, the plates were soaked in 96%, 90%, 80%, 70% alcohol for three minutes each, before being rinsed with running water for 5 minutes. After drying, the plates were immersed in hydrogen peroxide (H_2_O_2_) for 5 to 10 minutes, then washed twice with phosphate buffer saline (PBS), each for 4 minutes. Drops of Ultra V block were added for 5 minutes before being rinsed. Tissue staining was subsequently performed by adding rabbit anti-BDNF polyclonal antibody (Bioss, Woburn, Massachusetts, USA) for 60 minutes and washing with PBS twice for 4 minutes. Following, Biotinylated link drops (yellow) were added for 30 minutes and washed with PBS twice for 4 minutes. DAB (3,3 diaminobenzidine) chromogen (Sigma, St. Louis, Missouri, USA) was added for 6 to 10 minutes and washed with PBS twice for 4 minutes. Counterstain was performed using hematoxylin meyer and the sections were mounted. The slides were examined using a light microscope at 400x magnification. To count BDNF expression, pictures of five different fields were randomly chosen and taken using a DS Fi2 300-megapixel digital camera and an image processor.^15^

### Statistical analysis

Collected data were analyzed using Kolmogorov-Smirnov test to find the distribution. Given that all variables were normally distributed, the resulting data were analyzed with the SPSS 16 software for Windows (SPSS Inc., Chicago, USA) using a one-way ANOVA test, and continued using Tukey's LSD test to find differences between each group. Results are presented as mean and standard deviation (SD). The significance level considered was 0.05.

## Results

**Figure 1:**
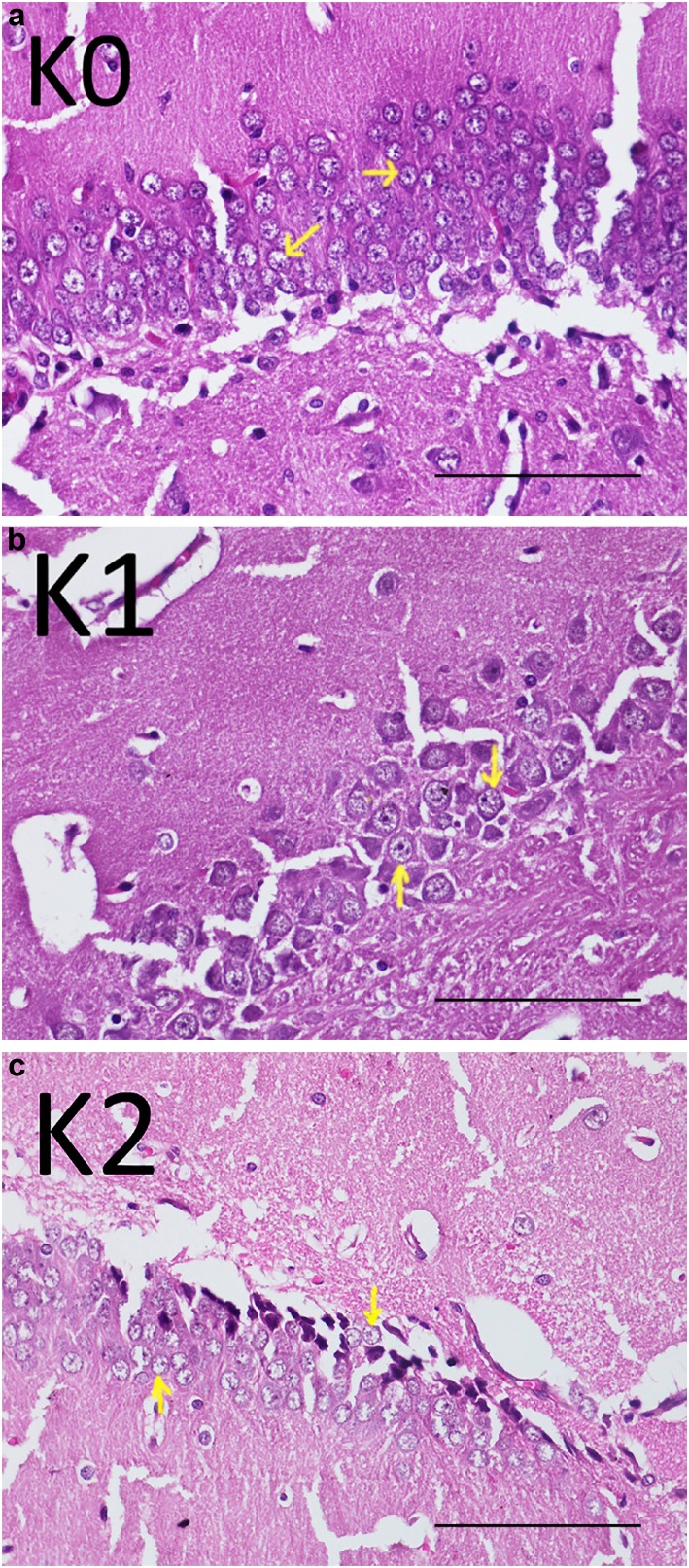
Hippocampus coronal section. Pyramid cells (arrow) of control group (K0) (a), soft diet group (K1) (b), and hard diet group (K2) (c)

Table 1 presents the results of pyramidal cell count and BDNF expression. Figure 1, which contains coronal section imaging of the hippocampus, shows that the control group had more pyramid cells in the hippocampus than the soft diet or hard diet groups. One-way ANOVA showed that there was a significant difference between the three groups (p=0.049; r^2^=0.285). Pyramid cell count of the K0 group was considerably higher when compared to those of K1 (p=0.036) and K2 (p=0.029) groups. Additionally, although K1 had more pyramid cells than K2, there was no significant difference (p=0.909).

**Table 1:** Pyramid cell count and BDNF expression (Mean±SD). Means with different superscript letters are statistically significant (Tukey's LSD, P<0.05)

Group	Pyramid Cell (mean±SD)	BDNF Expression (mean±SD)
K0	169.14^a^±27.25	85.27^a^±19.78
K1	130.14^b^±29.32	49.57^b^±20.90
K2	128.14^b^±39.02	36.86^b^±28.97

**Figure 2:**
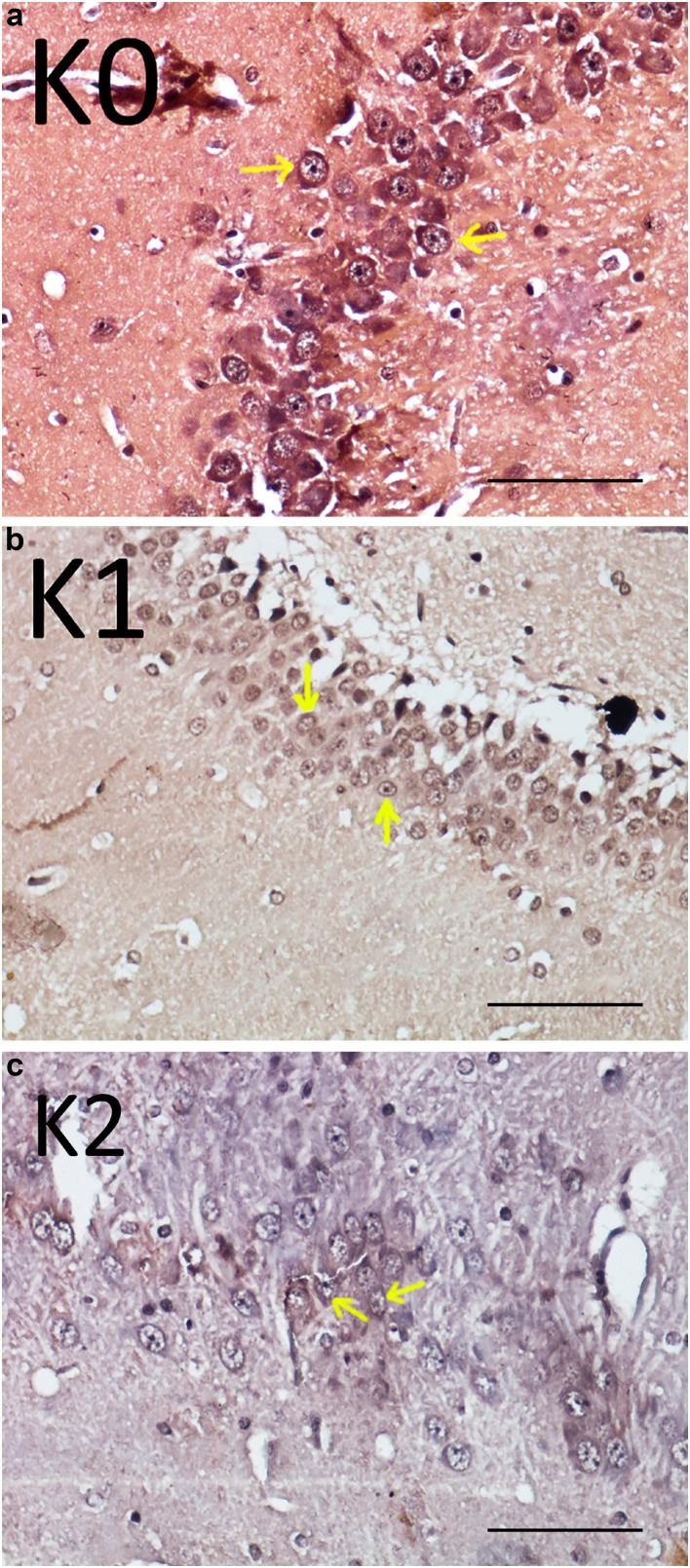
Hippocampus coronal section. Pyramid cells (arrow) that express BDNF (arrow) in K0 (a), K1 (b), and K2 (c)

Regarding BDNF expression, observation results were similar to pyramid cell count results. BDNF expression in K0 was higher than in K1 and K2 groups (Figure 2). The respective expression within groups was significantly different (p=0.006; r^2^=0.438). BDNF expression in K0 was significantly higher when compared to K1 (p=0.017) and K2 (p=0.002). Furthermore, BDNF expression in the K1 group was slightly higher than in the K2 group, but no significant difference between these groups (p=0.326) was identified.

## Discussion

This study was conducted using animal models with contrasting chewing habits to differentiate the respective masticatory intensities of each group. Post-weaned rats were used to facilitate the examination of the response regarding different chewing intensities as rapidly as possible. Sensory input into periodontal mechanoreceptors, resulting from masticatory force, functions as a feedback mechanism in regulating muscle contraction.^18^ Previous studies have assessed hippocampus function in rats’ growth based on the provision of food with varying hardness; thus, this research was performed to attest the results of the previous study by observing hippocampus function and morphology through cellular and molecular aspects.^14^

In this study, the K0 group had the highest number of pyramid cells and greater BDNF expression when compared to the K1 and K2 groups. However, K2 showed higher results when compared to K1, was although with no significant difference. According to the results of this study, a long-term soft diet led the same results as that of a number of previous studies, which argued that a long-term soft diet may reduce pyramid cell counts and BDNF expression in the hippocampus.^7^ ^,^ ^19^ Interestingly, the hard diet group showed a different result from the previous study, which stated that enforced mastication can increase neuron survival and Neural Stem Cell (NSC) activity in the dentate gyrus of the hippocampus; thus, potentially increasing learning and memory function.^12^ However, the cited study was performed on older animals when compared to this study. The post-weaned rats used in this study were in the transition period between suckling and using mastication for feeding.^20^ ^,^ ^21^

The result obtained is in accordance with our previous one, which assessed hippocampus function using behavior tests. The study found that the normal diet group had the best performance in behavior tests, followed by soft diet and hard diet groups. This showed that behavior assessment may represent the histological structure of hippocampus.^14^ These findings also corroborate to another study by Okihara, et al.^7^ (2014), which found a memory impairment accompanied by low number of neuron cells in the hippocampus after long-term soft diet.^7^ Previous studies on mastication impaired animal models through tooth extraction^22^ ^,^ ^23^, bite-raise^24^ ^,^ ^25^ and long-term soft diets^7^ ^,^ ^26^ found that these aspects may alter hippocampus learning and memory function. Other studies also proved that impaired function of the hippocampus due to long-term soft diet could be improved by providing a hard diet.^27^ ^,^ ^28^ Therefore, mastication activity is critical in maintaining hippocampus function for learning and memory.^29^ ^,^ ^30^

Newborn rats obtain nutrition supplies from breast-feeding alone, without any other food or water, until they are 14 days old. The weaning process begins at that point, with the breast-feeding period being completely finished at 30 days of age. During the weaning period, the transition from suckling to mastication and an increase in the jaw closing muscle activity also occurs. Thus, firing frequency of the motor neuron within the jaw closing muscle will continuously intensify as newborns develop to prepare the muscle for the mastication process.^20^ Other studies also argued that physiological adaptation of an organ will increase with growth. Thus, it is possible that the response to different mastication loads will differ according to age.^21^ Given that the samples in this study were all in the transition period from suckling to chewing movement, we can assume that this is the main cause for the low pyramid cell count and BDNF expression in hippocampus of hard diet group. Rats in the transition period may need adaptation to chewing movements; thus, increasing the food hardness gradually is necessary according to growth requirements.

Mastication activity constitutes the combination of motor functions that consist of jaw, tongue, mouth and cheek movements. Such movement pattern changes continuously in response to sensory feedback from the oral cavity and is formed according to the hardness and texture of the digested food. Chewing activity in rats starts to develop at the end of the second postnatal week, when their teeth emerge, although not being dependent on the tooth emergence process. In rats, the mastication movement is controlled by the cerebral cortex, more specifically the Central Pattern Generator (CPG), located in the brain stem, on the other hand, the suckling movement does not require control from the CPG.^31^

Rats possess a circuit that controls the basic pattern of jaw movement within the mastication process, being located in the brain stem that belongs to the main trigeminal sensory nucleus (NVsnpr) and its dorsal section is expected to control masticatory rhythm. The neuron membrane in the dorsal section of NVsnpr will continue to grow during the first three postnatal weeks, especially during the transition period from suckling to chewing. Neuronal firing also occurs during this period. Immature neuron cells show superior adaptive and signaling ability in the first two postnatal weeks.^13^

The main limitation of this study was providing food of differing hardness but with the same nutritional content. Thus, this study used standard rodent chow, which is softer when compared to whole grain and seeds yet harder than powdered grain and seeds, as a control. However, the nutritional contents of each type are slightly different.

## Conclusion

During the growth period, reduced mastication due to a soft diet and forced mastication resulting from a hard diet have been proven to reduce pyramid cell counts and BDNF expression in the hippocampus. A standard diet produced the optimum effect on the hippocampus. The degree of hardness of the food to be chewed should be well-suited to stages of development, in this case, hippocampus development in post-weaned period.

### Recommendation

This study used different types of food with the control and treatment groups. Given the limitations of this study, to obtain a deeper understanding, further research using the same kind of food of various hardness is required.
